# Effects of daily use of intermittent pneumatic compression in competitive handball players: A randomized controlled trial

**DOI:** 10.5114/biolsport.2025.151656

**Published:** 2025-06-24

**Authors:** Sergi Nuell, Jordi Rabassa, Cristina Bárcena, Josep Espar, Carles Munné, Iker García

**Affiliations:** 1 EUSES University School of Health and Sports, University of Girona, Salt, Spain; 2 Department of Physical Activity Sciences, Faculty of Education, Translation, Sports and Psychology, University of Vic – Central University of Catalonia, Vic, Spain; 3 Department of Cell Biology, Physiology and Immunology, Faculty of Biology, University of Barcelona, Barcelona, Spain

**Keywords:** Intermittent sequential pneumatic compression, Blood pressure, Pressotherapy, Tensiomyography, Team-sport performance, Muscle stiffness

## Abstract

Intermittent sequential pneumatic compression (ISPC) is used to improve readiness and recovery in athletes. This study aimed to evaluate the effects of daily use of ISPC for 5 weeks on the performance, physiological, and psychological parameters in seventeen male handball players. Players were randomly assigned either to an experimental (EXP, n = 8) or a control (CON, n = 9) group. Before and after the intervention, we measured the countermovement jump (CMJ) and the agility test (T-test) as markers of sport-specific performance, the systolic blood pressure (SBP) in the brachial and ankle arteries to evaluate the hemodynamic function, and the tensiomyography of biceps femoral, gastrocnemius and vastus medialis to assess muscle function. During the intervention, the session rating of perceived effort (sRPE, 30 minutes after training) and perceived recovery status (PRS, 1 hour after waking-up the following morning) were registered to evaluate subjective recovery. Results showed that CON experienced a decrease in agility performance from pre- to post-intervention (p = 0.030). In contrast, EXP had a significant improvement in the muscle contraction delay time of the left biceps femoris (p = 0.002), and a significant decrease in ankle SBP after the intervention (p = 0.017). Regarding perceived fatigue and recovery, EXP had slightly higher values than CON in PRS (p = 0.047), while sRPE had no significant changes. Thus, daily use of intermittent pneumatic compression for 5 weeks during a training period slightly mitigates the fatigue-induced effects of training, while enhancing hemodynamic regulation and subjective recovery in competitive handball players.

## INTRODUCTION

Handball is a team sport characterized by intermittent and multidirectional high-intensity actions and frequent body contact interspersed with incomplete recovery periods [1, 2]. Players need a high degree of aerobic and anaerobic fitness in addition to high levels of strength [2]. Moreover, the competition system with a short offseason and a long in-season featuring plenty of matches once or twice a week places high stress on the handball player, making recovery one of the most important aspects [1–3].

Recovery is a complex and multifactorial physiological and psychological process related to time, which is intended to restore exercise-induced fatigue [3, 4]. Post-exercise recovery is one of the main aspects to consider in athletic training due to its impact on sports performance and injury risk prevention [3–5]. There are several post-exercise recovery methods used for athletes, with passive mechanical strategies being a relevant trend in recent years [6]. Intermittent sequential pneumatic compression (ISPC) consists of a rhythmic gradual pressure on the limbs, which is applied to facilitate lymph and blood flow potentially enhancing the recovery after exercise [3]. Besides, ISPC may show a broad range of effects upon different compression magnitude, modes, and frequency of the compression-decompression [3, 6].

Currently, research into the effects of ISPC on athletic performance is limited to acute interventions, without considering the effects of a prolonged use of passive mechanical strategies. Acute interventions have shown positive results in regulating physiological homeostasis [7–9] and perceived recovery [3, 8, 10]. However, the use of ISPC has demonstrated limited results in improving muscular function or acutely reducing muscle damage markers after exercise [3, 9–11].

Muscle function is usually quantified by maximal voluntary contraction, which is considered a reliable indicator of physical fatigue and allows the specific assessment of central and peripheral subcomponents of fatigue [12, 13]. Surface mechanomyographic methods have been developed to monitor the acute effects of muscle mechanical properties without causing additional fatigue [12, 14]. Tensiomyography (TMG) is a non-invasive technique used to evaluate muscle function by recording the contractile response of a muscle to an electrical stimulus. This system assesses different parameters extracted from its waveform after a percutaneous neuromuscular stimulation and obtaining a displacement-time curve [12, 14]. According to the evidence, alteration of the TMG parameters has been related to increased muscle stiffness, less activation of the muscle fibers, muscle fatigue, and exercise-induced muscle damage [12, 14–16].

During exercise, blood flow increases through the arteries creating frictional force, or wall shear stress along the endothelium [17]. A similar mechanism has been described when applying external counterpulsation [18], potentially benefiting lymph and blood flow. Brachial and ankle systolic blood pressure (SBP) measure, respectively, systemic and regional hemodynamic function. When used acutely, ISPC has been shown to improve post-exercise recovery of heart rate, SBP, and peripheral vascular resistance [7], as well as blood hematocrit [8], but the potential benefits for athletes at the vascular level have not been studied in prolonged interventions.

ISPC devices are designed to promote recovery; however, most of the studies on ISPC focus on acute interventions, even though the potential effects of their prolonged use remain unclear. Further research is needed to shed some light on their long-term use, especially in ecological environments. Therefore, this study aimed to evaluate the effects of daily use of ISPC for 5 weeks on the recovery of competitive handball players. We evaluated the sport-specific performance and readiness through countermovement jump (CMJ) and the agility T-test; the physiological status through brachial and ankle SBP and TMG parameters; and the perceived fatigue and recovery through the session rating of perceived effort (sRPE) and the perceived recovery status (PRS). We hypothesized that using ISPC daily for 5 weeks would have a significant impact on the athlete’s recovery, allowing them to mitigate fatigue as well as increase performance and physiological readiness.

## MATERIALS AND METHODS

### Participants

**FIG. 1:**
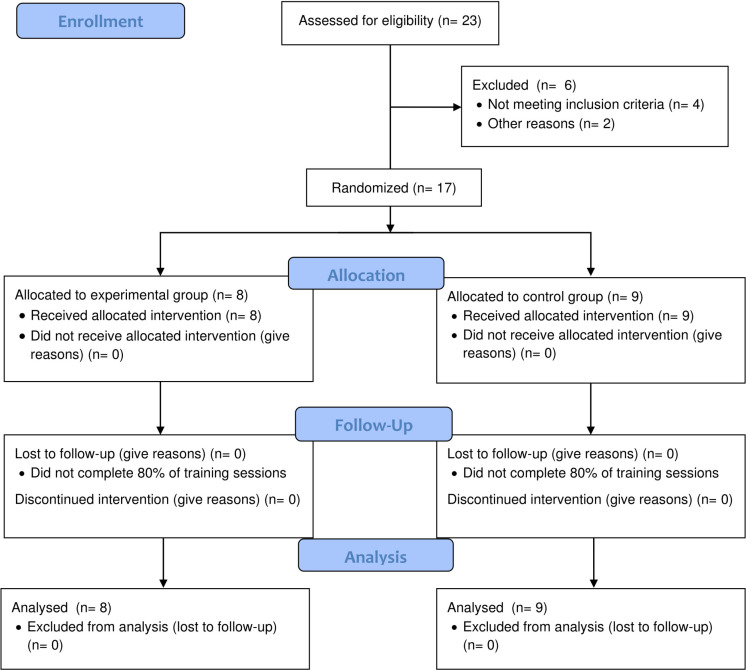
CONSORT flow diagram of participants through the trial.

Seventeen national-level male handball players volunteered to participate in the study. Participants were randomly assigned to one of two groups, experimental (EXP) or control (CON). The EXP group was composed of 8 players (age: 21.1 ± 2.9 y, height: 180.9 ± 6.4 cm, weight: 86.7 ± 11.5 kg), while the CON group was composed of 9 players (age: 21.5 ± 4.4 y, height: 182.0 ± 5.9 cm, weight: 81.7 ± 11.4 kg) (Figure 1). All participants were adequately informed, participated voluntarily, and signed a consent form (parents or guardians in the case of those under 18 years of age).

All participants had more than 10 years of handball training experience, performing three training sessions of handball per week (6 hours) plus the weekend handball match during the study. The athletes can be categorized as highly trained / national level (Tier 3) [19]. All of them were part of the same training group with similar training methodology, routines, volumes, intensities, and loads. Goalkeepers were excluded from the study to keep the training load as similar as possible between all the participants.

All procedures were approved by the Institutional Review Board (IRB00003099: CER022441) at the University of Barcelona, the study was conducted in accordance with the Declaration of Helsinki and all participants provided written informed consent before the start of the study.

### Experimental design

**FIG. 2:**
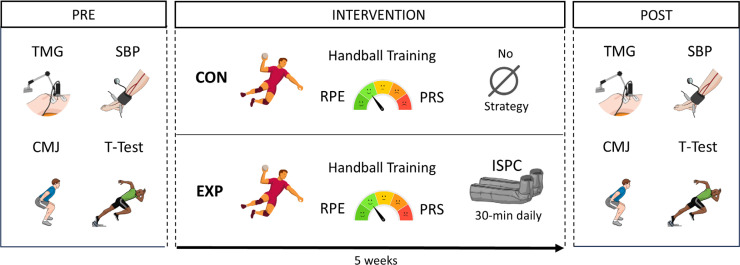
Experimental design of the study. Note: TMG (Tensiomyography); SBP (Systolic Blood Pressure); CMJ (Countermovement Jump); T-Test (Agility T-test); CON (Control group); EXP (Experimental group); RPE (Rate of Perceived Exertion); PRS (Perceived Recovery Status); ISPC (Intermittent Sequential Pneumatic Compression).

Participants were tested (in the afternoon) at the same time of the day to avoid potential diurnal variations. Before the measurements, subjects abstained from caffeine, fasted for 4 h, and abstained from exercise for at least 48 h. To study the effects of ISPC on the handball players’ sports physiology and performance (Figure 2), an initial evaluation (PRE) was done before the beginning of the study. The evaluation consisted of brachial (systemic) and ankle (regional) SBP, a TMG evaluation, and sport-specific performance tests (CMJ and agility T-test). Two familiarization sessions were performed to educate the correct sequence of the sport-specific performance tests before the beginning of the study. During the intervention, participants reported the sRPE of each training session and PRS on the following day through online self-reporting using XPS Network APP. Five weeks after the intervention, there was a second evaluation (POST) following the same procedures as described in PRE.

Participants in the EXP group were asked to perform unsupervised and home-based ISPC for 5 consecutive weeks, 7 times per week, and 30 minutes per session, either at the end of the afternoon training or competition, or at the end of the day (on resting days). The length of the experiment was based on team availability, considering 5 weeks and daily use of ISPC (up to a total of 750 minutes) enough to affect physiological and performance parameters in competitive handball players.

### Procedures

#### Intermittent sequential pneumatic compression

An intermittent sequential pneumatic compression device (Recovery Air 3 PRO, Therabody®, Los Angeles, CA) was used for ISPC intervention. This pneumatic compression device consists of 2 separate “leg sleeves”, which contain 4 circumferential inflatable chambers (arranged linearly along the limb) encompassing the leg from the feet to the hip/groin. The “leg sleeves” are connected to an automated pneumatic pump at which target inflation pressures for each zone and the duty cycle can be controlled. The compression modality was sequential, where a single pressure is applied to parts of the limb in a distal-to-proximal sequence with 4 overlapped chambers per sleeve, creating a negative pressure gradient from distal to proximal with a continuous decline of 1 mmHg in the subsequent proximal cells.

The duration of the session was based on Artes et al. [7] who described post-exercise acute cardiovascular modulation using ISPC. The selected pressure increased progressively throughout the intervention, from 85 (1^st^ week) to 90 (2^nd^–3^rd^ week) and 95 (4^th^–5^th^ week) mmHg. This is slightly higher pressure than previous studies demonstrating an enhancement of athletic recovery in acute singleday session [3, 7]. The level of compression increased biweekly to avoid physiological accommodation. Additionally, the participants were instructed not to use other recovery strategies, such as coldwater immersion, stretching, or foam rolling for the 5 weeks.

### Countermovement jump

A CMJ test was performed to assess lower-body explosive strength, as well as to assess readiness and fatigue [5, 13]. Participants began in a tall standing position, with feet placed shoulder-width apart on a contact platform (Chronojump Boscosystem®, Barcelona, Spain). Then, participants dropped into the countermovement position to a self-selected depth, followed by a maximal-effort vertical jump. Hands remained on the hips for the entire movement to eliminate any influence of arm swing and ankles landed in a full extension. Each participant completed three to five trials of the CMJ until a plateau in the jump height was detected, with a 1 min rest period between each trial. The average of the three best jumps, based on the jump height, was used for further analysis [20].

### Agility T-test

**FIG. 3:**
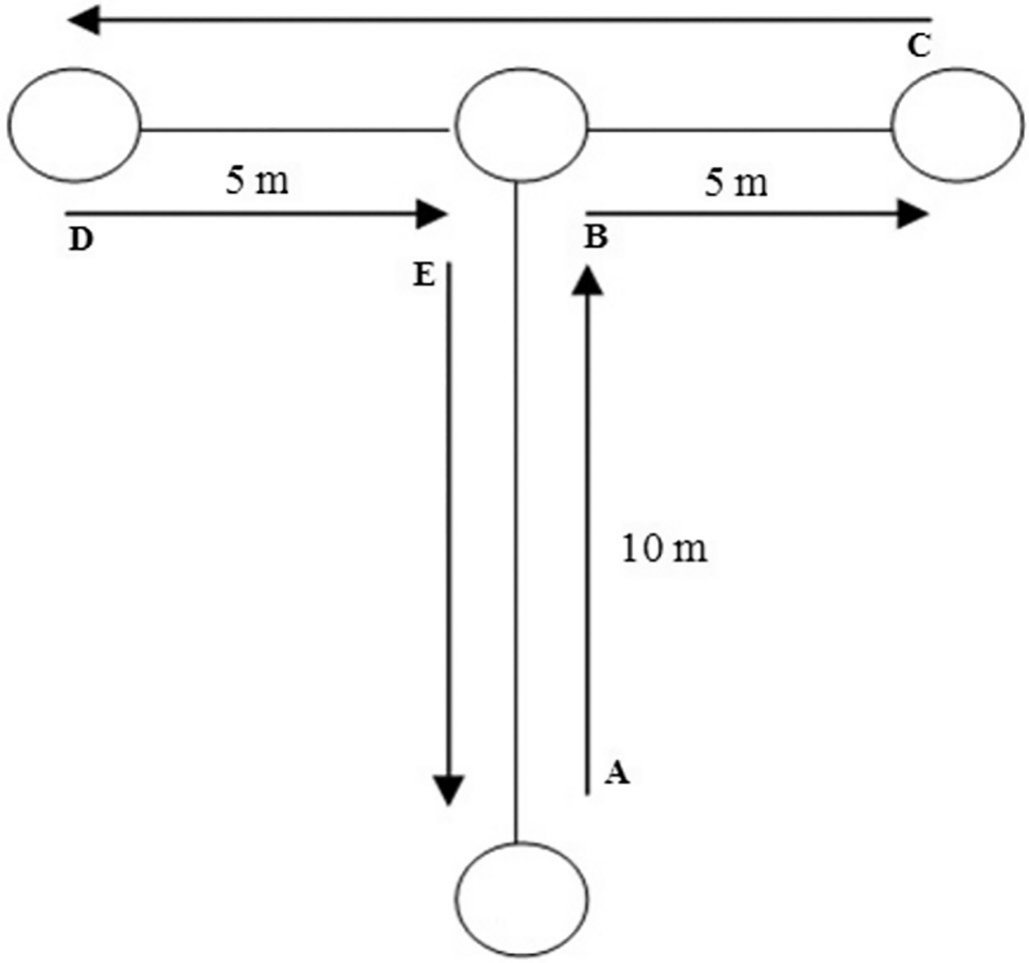
Modified Agility T-test. Note: Participants sprinted 10 m straight and touched the tip of the cone (B), then shuffled 5 m to the right side and touched the tip of the cone (C). Next, participants shuffled to the left side 10 m and touched the tip of the cone (D), immediately shuffled 5 m to the right side and touched the tip of the cone B. Finally, participants ran backward until they passed the starting line (A).

The modified T-test assesses multidirectional movement ability including speed, agility, and quickness [21]. Participants sprinted forward, backwards and shuffled right and left as depicted in Figure 3. If participants crossed their legs on the lateral shuffle or they did not touch the cone, the attempt was repeated. Test time was recorded using a pair of photocells (Witty, Microgate, Bolzano, Italy) placed at the start line to measure total time. Participants were placed right before the starting line with a two-point standing start, and they were told to start without any countermovement at will. Each participant performed two maximal effort attempts with 5 min of rest between them. The best time was retained for analysis.

### Tensiomyography

Maximum muscle radial displacement of the muscle belly (Dm), contraction time (Tc), and delay time (Td) of the biceps femoris (BF), gastrocnemius medialis (GM), and vastus medialis (VM) from both legs were assessed using a TMG system (TMG measurement system, Ljubljana, Slovenia). Dm represents the maximal radial displacement of the muscle belly (mm) [12, 14]. Tc represents the time (ms) the muscle needs to reach its maximum displacement after receiving the electrical stimulus, and Td represents the time (ms) the muscle needs to begin its contraction after receiving the electrical stimulus [12, 14].

Participants were asked to remain relaxed in a rest position for 5 min. For the BF and GM evaluation, the knee was fixed at 175° and the ankle was free outside the stretcher. When evaluating VM, participants were in a supine position and a pad was used to fix the knee at 160°. Electrodes (Compex Medical AS, Ecublens, Switzerland) were placed at a 5 cm distance from each other avoiding the tendon insertions [12]. The measurement point was set at the maximal radial circumference of each muscle. Electrodes were connected to an electrical stimulator (TMG-S2 doo, Ljubljana, Slovenia). A digital displacement transducer (GK 40, Panoptik doo, Ljubljana, Slovenia) set at 0.17 N · m^-1^ was positioned perpendicular to the previously established measurement point of the muscle belly [12, 14]. The protocol started at 40-mA, increasing by 30 mA until the maximal radial displacement was obtained, separated by 10-s rest [12].

### Systolic blood pressure

Brachial and ankle SBP were recorded using a standard technique [22]. After a 5-minute supine rest position with straight arms and legs, duplicate SBP measurements were recorded in both upper (brachial artery) and lower extremities (dorsalis pedis and posterior tibial arteries), and the average of each pair of readings was recorded. In the case of ankle SBP, the higher average values from the dorsalis pedis or posterior tibial were considered [22].

Brachial SBP was taken using an automated oscillometric device (OMRON BP785, Omron Corporation, Kyoto, Japan). The cuff was positioned at the axillary midline portion on the brachial artery, with the lower edge of the cuff positioned 2–3 cm above the antecubital fossa for the arm. Ankle SBP was measured using a hand-held Doppler ultrasound device (Hi-dop BT-200V, Bistos Co Ltd, Seongnam, South Korea) operated with an 8-MHz probe and an aneroid manual sphygmomanometer (Riester minimus® III, Rudolf Riester GmbH, Jungingen, Germany).

The probe was placed in the area of the pulse at a 45° to 60° angle to the skin. The ankle cuff was placed just above the malleoli and was inflated up to 20 mmHg above the level of flow signal disappearance. Thereafter, the cuff was slowly released until the flow signal returned. The blood pressure that coincides with the return of the flow signal was considered the ankle SBP [23].

### Session rating of perceived exertion

The methodology proposed by Foster et al. [24] was used to calculate the sRPE. Thirty minutes after ending every training session or competition players were individually asked about their perceived effort during the training or competition using the modified Borg scale of 1 to 10, 1 being no effort and 10 maximum effort.

### Perceived recovery status

The players’ PRS was assessed the day after each training session and competition, 1 hour after waking up, using a modified 10-point recovery scale [25]. The players were asked to indicate the degree of perceived recovery based on psychophysical cues (i.e., muscle soreness, tiredness, and mood states), being 1 not recovered at all and 10 indicating maximally recovered.

### Statistical analysis

Data are reported as mean ± SD. First, a Shapiro–Wilk test was used to establish the normal distribution of the sample, and the Levene test was used to assess for homogeneity of variance between groups. Differences in the studied parameters between pre- and post-intervention conditions were analyzed using a 2-way repeated-measures analysis of variance with 2 independent factors: group inter-subjects (EXP vs CON) and time intra-subjects (PRE vs POST). In the case of detecting statistical effects (*p* value < 0.05), post-hoc Holm-Bonferroni comparisons were performed. The effect sizes are expressed as partial eta-squared ($\eta_{\text{p}}^{2}$) for the repeated-measures ANOVA, and as Cohen’s *d* for pairwise comparisons. The magnitude of effect for $\eta_{\text{p}}^{2}$ was interpreted as follows: $\eta_{\text{p}}^{2}$ ≥ 0.01, *small* effect; $\eta_{\text{p}}^{2}$ ≥ 0.06, *medium* effect; and $\eta_{\text{p}}^{2}$ ≥ 0.14, *large* effect. Cohen’s *d* was interpreted as follows: *d* ≥ 0.1, ≥ 0.3, ≥ 0.5, ≥ 0.7, and ≥ 0.9 reflecting *small, moderate, large, very large*, and *extremely large* effect sizes, respectively [26]. All analyses were run using SPSS (version 29, IBM SPSS Statistics) and R (version 4.4.2) in RStudio.

## RESULTS

### Performance

**FIG. 4:**
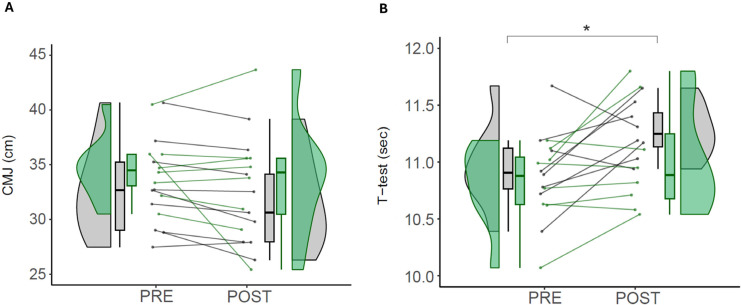
Sport-specific performance. Note: Values of countermovement jump test (A) and agility T-test (B) in control (CON) and experimental (EXP) groups before and after the intervention. CMJ (Countermovement Jump). CON are denoted in gray, while EXP are denoted in green. * denotes statistically significant differences from pre- to post in the CON group (p < 0.05).

Triplicate measures of CMJ show a test-retest intraclass correlation between conditions (ICC 3, 1) of 0.921. The CMJ measurements were not statistically significantly different between EXP and CON in the time*group comparison (*p* = 0.961). Within groups, there were no effects from pre- to post-intervention in EXP (*p* = 0.332) or CON (*p* = 0.336) (Figure 4A).

Duplicate measures of agility T-test show a test-retest intraclass correlation between conditions (ICC 3, 1) of 0.795. In the group*time interaction, there were no statistically significant differences between EXP and CON (*p* = 0.603). In the comparisons within groups, there were significant differences from pre- to post-intervention in the CON (10.95 ± 0.37 vs 11.27 ± 0.24 sec; *p* = 0.030; *d* = 1.03), but not in the EXP (10.80 ± 0.36 vs 11.02 ± 0.47 sec; *p* = 0.120; *d* = 0.53) (Figure 4B).

### Tensiomyography

In the group*time interaction, there were no statistically significant differences between EXP and CON (*p* > 0.099; $\eta_{\text{p}}^{2}$ < 0.184). In the CON, no significant changes were observed between the pre- and post-intervention in any of the variables. However, in the EXP, there were differences in the Td (23.43 ± 4.27 vs. 21.36 ± 2.94 ms; *p* = 0.003; *d* = -0.57), Tc (26.92 ± 7.25 vs. 23.75 ± 8.78 ms; *p* = 0.167; *d* = -0.39) and Dm (3.06 ± 1.02 vs. 2.80 ± 1.45 ms; *p* = 0.613; *d* = -0.21) of the left BF; and in the Tc (19.30 ± 3.72 vs. 19.00 ± 2.83 ms; *p* = 0.918; *d* = -0.09), Td (21.61 ± 2.60 vs. 19.10 ± 2.24 ms; *p* = 0.434; *d* = -1.03) and Dm (2.44 ± 0.81 vs. 1.90 ± 0.90 ms; *p* = 0.184; *d* = -0.63) of the right GM (Table 1).

**TABLE 1:** Changes in tensiomyography parameters in the experimental and control groups.

	EXP					CON				

	Pre	Post	*p*-value	*d*	CI (95%)	Pre	Post	*p*-value	*d*	CI (95%)
Biceps Femoris (right)	Tc (ms)	20.80 ± 4.01	22.83 ± 8.15	0.394	0.32	-0.67; 1.302	26.05 ± 7.96	28.18 ± 7.52	0.345	0.28	-0.653; 1.203
	Td (ms)	21.40 ± 1.76	21.41 ± 2.91	0.992	0.00	-0.976; 0.984	24.22 ± 8.34	23.32 ± 4.67	0.440	-0.13	-1.058; 0.792
	Dm (mm)	2.65 ± 1.07	2.77 ± 1.19	0.819	0.11	-0.875; 1.087	2.41 ± 0.63	2.55 ± 1.03	0.779	0.16	-0.762; 1.089

Biceps Femoris (left)	Tc (ms)	26.92 ± 7.25	23.75 ± 8.78	0.167	-0.39	-1.383; 0.596	27.80 ± 8.16	29.91 ± 10.74	0.322	0.22	-0.706; 1.148
	Td (ms)	23.43 ± 4.27	21.36 ± 2.94 *	**0.002**	-0.57	-1.564: 0.435	24.01 ± 5.08	22.90 ± 4.50	0.053	-0.23	-1.158; 0.696
	Dm (mm)	3.06 ± 1.02	2.80 ± 1.45	0.613	-0.21	-1.19; 0.775	2.65 ± 0.82	2.71 ± 0.82	0.909	0.07	-0.851; 0.997

Gastrocnemius Medialis (right)	Tc (ms)	19.30 ± 3.72	19.00 ± 2.83	0.918	-0.09	-1.139; 0.957	18.27 ± 3.83	22.30 ± 11.61	0.132	0.47	-0.47; 1.403
	Td (ms)	21.61 ± 2.60	19.10 ± 2.24	0.434	-1.03	-2.15; 0.081	20.16 ± 2.92	23.38 ± 10.53	0.261	0.42	-0.517; 1.351
	Dm (mm)	2.44 ± 0.81	1.90 ± 0.90	0.184	-0.63	-1.704; 0.443	1.77 ± 1.45	2.04 ± 1.36	0.449	0.19	-0.734; 1.118

Gastrocnemius Medialis (left)	Tc (ms)	21.84 ± 6.34	24.67 ± 7.11	0.240	0.42	-0.639; 1.479	18.47 ± 3.04	22.45 ± 8.52	0.071	0.62	-0.324; 1.568
	Td (ms)	22.10 ± 1.57	20.68 ± 1.64	0.395	0.50	-0.565; 1.563	20.35 ± 2.21	21.56 ± 5.57	0.409	0.29	-0.643; 1.214
	Dm (mm)	2.60 ± 1.00	2.45 ± 1.08	0.649	-0.14	-1.193; 0.905	1.84 ± 1.38	2.03 ± 1.22	0.497	0.15	-0.779; 1.071

Vastus Medialis (right)	Tc (ms)	23.41 ± 3.49	24.62 ± 3.02	0.495	0.37	-0.618; 1.359	26.15 ± 5.25	25.51 ± 1.37	0.718	-0.17	-1.149; 0.815
	Td (ms)	20.03 ± 2.15	20.06 ± 1.81	0.969	0.02	-0.965; 0.995	20.85 ± 0.91	20.67 ± 1.27	0.784	-0.16	-1.145; 0.819
	Dm (mm)	5.01 ± 1.99	5.01 ± 1.78	1.000	0.00	-0.98; 0.98	6.47 ± 1.06	6.08 ± 1.04	0.523	-0.37	-1.36; 0.617

Vastus Medialis (left)	Tc (ms)	25.46 ± 7.68	25.47 ± 2.84	0.994	0.00	-0.978; 0.982	25.85 ± 4.13	26.16 ± 1.96	0.852	0.10	-0.885; 1.076
	Td (ms)	20.75 ± 2.14	20.10 ± 1.32	0.261	-0.37	-1.354; 0.623	21.68 ± 1.47	20.87 ± 1.09	0.165	-0.63	-1.63; 0.378
	Dm (mm)	5.12 ± 2.01	5.18 ± 2.01	0.900	0.03	-0.95; 1.01	6.52 ± 0.97	6.02 ± 1.44	0.324	-0.41	-1.397; 0.583

EXP (Experimental group); CON (Control group); Tc (Contraction Time); ms (milliseconds); Td (Delay Time); Dm (Muscle Displacement); mm (millimetres); *d* (Cohen’s *d*); CI (Confidence Interval). Values are means ± SD.

* denotes statistically significant differences from Pre- to Post-Intervention.

### Systolic blood pressure

Baseline duplicate measures of SBP show a test-retest intraclass correlation between conditions (ICC 3, 1) of 0.928. In the group*time interaction, there were no differences between EXP and CON in brachial SBP (*p* = 0.383) and ankle SBP (*p* = 0.389).

**FIG. 5:**
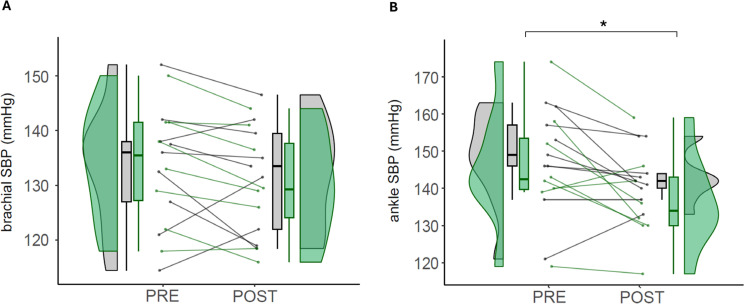
Vascular function. Note: Values of brachial SBP (A) and ankle SBP (B) in control (CON) and experimental (EXP) groups before and after the intervention. SBP (Systolic Blood Pressure). CON are denoted in gray, while EXP are represented in green. * denotes statistically significant differences from pre to post in the EXP group (p < 0.05).

Pairwise comparison for brachial SBP shows that there were no statistically significant differences in any group between pre- and postintervention, neither EXP (134.13 ± 10.76 vs. 130.06 ± 10.03 mmHg; *p* = 0.075; *d* = −0.39) nor CON (133.39 ± 11.28 vs. 131.94 ± 10.18; *p* = 0.481) (Figure 5A).

Regarding ankle SBP, pairwise comparison reveals that EXP had a statistically significant decrease from the pre- to the post-intervention (145.88 ± 16.05 vs. 136.50 ± 12.60 mmHg; *p* = 0.017; *d* = −0.65), while CON had no statistically significant differences (148.22 ± 13.16 vs. 143.11 ± 10.28; *p* = 0.142; *d* = −0.43) (Figure 5B).

### Perceived effort and recovery

**FIG. 6:**
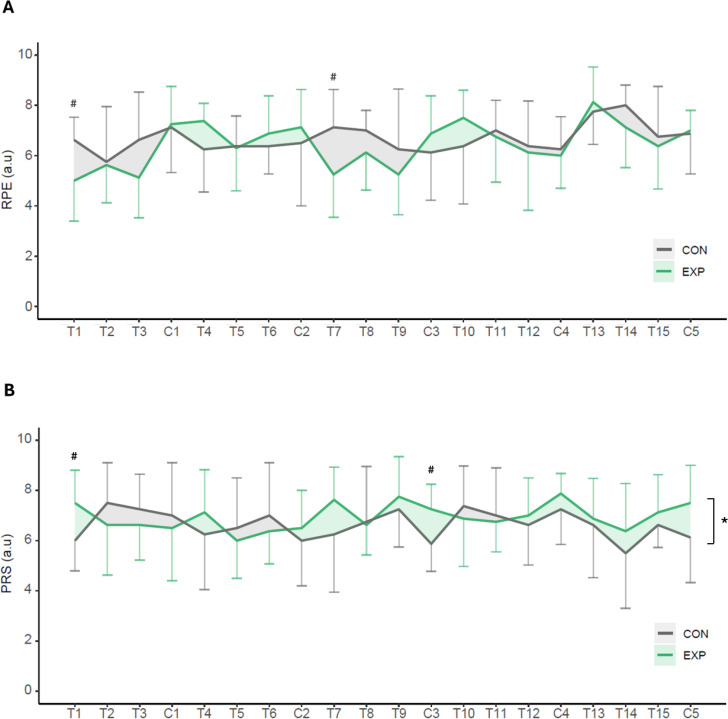
Perceived effort and recovery. Note: Time-course changes in perceived effort (A) and recovery (B) through the intervention in control (CON) and experimental (EXP) groups. Data points represent Mean, while error bars represent SD. RPE (Rate of Perceived Exertion); PRS (Perceived Recovery Status), T (Training) and C (Competition). * denotes statistically significant differences in the group*time interaction (p < 0.05). # denotes statistically significant differences between groups in a single time point (p < 0.05).

The time-course analysis of the sRPE shows a statistically significant increase throughout the intervention (F_19,266_ = 3.05; *p* < 0.001; $\eta_{\text{p}}^{2}$ = 0.179). In the group*time interaction, there were no differences between EXP and CON (F_19,266_ = 1.62; *p* = 0.053; $\eta_{\text{p}}^{2}$ = 0.103). Pairwise comparisons show that sRPE was significantly lower for the EXP on day 1 (5.0 ± 1.6 vs. 6.6 ± 0.9 a.u.; *p* = 0.026; *d* = 1.23) and 9 (5.3 ± 1.7 vs. 7.1 ± 1.5 a.u.; *p* = 0.031; *d* = 1.12), but there were no differences in the multivariate analysis for EXP (*p* = 0.785) and CON (*p* = 0.159) (Figure 6A).

The time-course analysis of the PRS shows no differences throughout the intervention (F_19,266_ = 1.59; *p* = 0.057; $\eta_{\text{p}}^{2}$ = 0.102). In the group*time interaction, there were statistically significant differences between EXP and CON (F_19,266_ = 1.64; *p* = 0.047; $\eta_{\text{p}}^{2}$ = 0.105). Pairwise comparisons show that PRS was statistically significantly higher for the EXP in the day 1 (7.5 ± 1.3 vs. 6.0 ± 1.2 a.u.; *p* = 0.031; *d* = −1.20) and 12 (7.3 ± 1.0 vs. 5.9 ± 1.1 a.u.; *p* = 0.023; *d* = −1.33), but there were no differences in the multivariate analysis for EXP (*p* = 0.732) and CON (*p* = 0.345) (Figure 6B).

### Post-hoc power analysis

A post-hoc power analysis calculation was computed. For a repeated measures ANOVA (within-between interaction), we considered the smallest effect size that yielded a statistically significant result (*d* = 0.57). With an α level of 0.05, and a total sample size of 17 participants, the output power (1 – β) of 0.992 (G*Power Version 3.1.9.7; Heinrich-Heine-Universität Düsseldorf, Düsseldorf, Germany).

## DISCUSSION

The present study aimed to evaluate the effects of the daily use of ISPC for 5 weeks on recovery, from a multifactorial perspective, on competitive handball players. The main findings after the intervention were: 1) agility performance worsened in the CON; 2) ankle SBP declined in the EXP; and 3) PRS was slightly higher in the EXP.

### Performance

Previous studies have yielded unsuccessful results of ISPC in improving athletic performance [8, 9]. However, most of the studies applied ISPC in an acute single-day intervention, lacking ecologically-based comparisons as this study does. To our knowledge, this is the first time that the effects of ISPC have been assessed in a longitudinal intervention. Still, the complex nature of athletic training and performance sets a permanent challenge when evaluating the results. This is relevant to interpreting the effects of technology-based interventions on physical performance.

Countermovement jump is a common performance assessment test, due to its simplicity, time efficiency, and the relevant kinematic movement [27]. After the intervention, we found no differences in CMJ in either EXP or CON, with a non-significant decrease in CMJ height in both groups. These findings are consistent with those of Northey et al. [28], who also observed no significant differences in CMJ after 45 minutes of ISPC at 80 mmHg. Similarly, Blumkaitis et al. [29] observed that the use of ISPC after an extenuating jumping protocol did not recover the CMJ height between the control and experimental groups. These results must be read with caution, since jump height may not be sensitive enough to assess neuromuscular status [27].

After the intervention, we found that EXP did not change the agility T-test performance, while CON suffered an *extremely large* decrease in performance. Taking the performance results together, the in-season training load produced an overall decrease in performance in both groups, although it was more noticeable in the CON, suggesting that the use of ISPC may attenuate the fatigue-induced effects of training.

### Muscle function

Regarding muscle mechanical function, we measured muscle Tc, Td, and Dm, which are the most frequently evaluated parameters and have the highest level of reliability [12, 14]. We found no clear overall differences between groups. For the BF muscle, we observed a *large* and significant reduction in Td, alongside *moderate* and *small* differences in Tc and Dm in the EXP but not in the CON, potentially indicating a decrease in neuromuscular fatigue as the muscle initiates contraction faster [12, 14–16]. For the GM muscle, we found a *very large* and a *large* reduction in Td and Dm, respectively, with no changes in Tc; alongside *small* to *moderate* increases in the TMG parameters of the CON, presumably as a response to a slower and more fatigated muscle [12, 14]. For the VM muscle, no differences were found between groups (Table 1).

Acute changes in TMG variables have been largely investigated and are related to exercise-induced fatigue [12, 16]. However longitudinal changes, like those of the present study, are less understood [11, 15, 21]. García-García et al. [11] evaluated professional football players before and after a 10-week training period and found decreases in Tc, Td, and Dm of knee extensors and increases in Dm of knee flexors, showing that TMG is sensitive to detect training changes, despite showing no clear results. García-Unanue et al. [21] found that elite futsal players were faster and more resilient compared to amateurs, but there were no differences in TMG parameters. When ISPC and cold-water immersions were combined to enhance recovery in team sports athletes, the authors found increases in Tc and Dm, as fatigue increased throughout the study [1]. However, in this study there was no control group, therefore further comparisons are limited.

### Vascular function

Ankle and brachial systolic pressure provide an indirect measurement of cardiovascular function, integrating cardiac workload, endothelial and hormonal activity [22]. Our results indicate that daily use of ISPC for 5 weeks reduces the ankle SBP (−9.38 mmHg, −6%), representing a *large* regional decrease of SBP in the area where it was implemented. One of the primary mechanisms of ISPC to improve vascular physiology is the rise of hemodynamic shear stress induced by high-pressure ISPC [30], which has been increased by 402% in the femoral artery of the lower limb during pneumatic compression synchronized to the cardiac cycle [18]. Besides, the frictional force along endothelial cell membranes (shear stress) is key for the release of nitric oxide from the endothelium when increased blood flow [31].

The time SBP returns to basal levels after exertion indicates the degree of recovery and physiological adaptation to training [32]. Some acute interventions describe a positive effect of the ISPC in different vascular parameters following a similar experimental design, including a sequential inflation mode from distal to proximal, and pressures higher than 70 mmHg. Martin et al. [33] found that a single 1-h bout of ISPC at 70 mmHg improved local resistance vessel reactivity (i.e. peak blood flow) and systemically improved flow-mediated dilation. Maia et al. [34] described that ISPC acutely increases blood flow in the femoral arteries, which may be consistent with delivering nutrients and removing metabolites from the active muscles. Aligned with our results, Artés et al. [7] reported improvements in cardiovascular recovery when using ISPC after high-intensity exercise, including SBP, cardiac output, and peripheral vascular resistance.

The present results may have relevant implications for performance and health preservation in clinical populations, since we described that prolonged use of ISPC decreased regional SBP in young athletes. A decrease in SBP may preserve the distensibility of the lumen of the arteries, decreasing the risk of cardiovascular events [35]. During aging, sustained increases in blood pressure promote matrix synthesis causing subsequent increases in vascular thickness and structural artery stiffening [36]. Our results show that ISPC decreased regional SBP (−9.38 mmHg), which is comparable to isometric exercise training (−8.24 mmHg) [37], and antihypertensive drugs (−10 to 15 mmHg), placing ISPC as a promising non-pharmacological approach.

### Perceived fatigue and recovery

Our analysis showed that sRPE was not different between groups during the intervention, while PRS had a slight increase in EXP, although results need to be interpreted with caution. The increased perceived recovery when using ISPC is aligned with other authors, who found that ISPC reduces subjective muscular fatigue scores acutely [8] and after 24 h [3, 8, 10] as well as a concomitant reduction of muscle tenderness and stiffness [38]. On the contrary, some studies showed only a trend towards a positive effect of the ISPC over the perceived recovery scale after an acute intervention with interval exercise [39, 40], and others did not obtain significant differences in perceived recovery [41].

Providing subjective ratings of recovery such as PRS when not blinded to the treatment is subject to potential placebo effects. The placebo effect has neurobiological underpinnings based on classical conditioning and expectancy which have actual effects on the brain and body [42]. We demonstrated a significant slight effect using ISPC daily for 5 weeks in competitive athletes, but the increases in perceived recovery with ISPC must be interpreted with caution in comparison with no recovery strategy.

### Limitations and future research

The duration of the study was adjusted to in-season team availability; a longer experimental design could provide further understanding of the effects of ISPC on athletic performance. Additional parameters such as arterial endothelial function and other mediators of vasodilation, muscle stiffness via shear-wave elastography, or autonomous nervous system regulation may describe in more detail the influence of the ISPC in the athletic environment.

Previous studies have suggested that using a placebo condition is required to assess the psychological or expectancy effects [32]. Comparing the experimental group with the control without a placebo may tease out the effects of the intervention through confounding biases such as participants’ expectations or the Hawthorne effect. A placebo intervention would have been required to mimic ISPC without delivering any actual physiological effect (sham-ISPC). This is a recognized limitation of this study which may be addressed in future studies with the inclusion of a placebo strategy without real effects in athletic recovery such as neutral cream with no recovery properties.

Additionally, the sample size used in the study is relatively small, although comparable to previous studies (7, 8). This demands caution when interpreting the results and highlights the importance of future research evaluating the “medium- and long-term” effects of ISPC (and other strategies) in post-exercise recovery.

The implication of prolonged use of technologically-based strategies to modulate physical fitness, health, and performance is unknown. Future research should focus on understanding whether there are potential risks in the prolonged use of these strategies.

## CONCLUSIONS

The more pronounced worsening in performance observed in the control group, the reduced SBP and the higher perception of recovery observed in the experimental group evidence that the daily use of ISPC in competitive handball players may mitigate the fatigueinduced effects of training while enhancing hemodynamic regulation and subjective recovery.
